# Barriers to surgery performed by non-physician clinicians in sub-Saharan Africa—a scoping review

**DOI:** 10.1186/s12960-020-00490-y

**Published:** 2020-07-17

**Authors:** Phylisha van Heemskerken, Henk Broekhuizen, Jakub Gajewski, Ruairí Brugha, Leon Bijlmakers

**Affiliations:** 1 Health Evidence Department, Radboud University Medical Centre, Nijmegen, The Netherlands; 2 Department of Public Health and Epidemiology, Royal College of Surgeons in Ireland, Dublin, Ireland

**Keywords:** Surgical task-shifting, Non-physician clinicians, Barriers

## Abstract

**Background:**

Sub-Saharan Africa (SSA) faces the highest burden of disease amenable to surgery while having the lowest surgeon to population ratio in the world. Some 25 SSA countries use surgical task-shifting from physicians to non-physician clinicians (NPCs) as a strategy to increase access to surgery. While many studies have investigated barriers to access to surgical services, there is a dearth of studies that examine the barriers to shifting of surgical tasks to, and the delivery of safe essential surgical care by NPCs, especially in rural areas of SSA. This study aims to identify those barriers and how they vary between surgical disciplines as well as between countries.

**Methods:**

We performed a scoping review of articles published between 2000 and 2018, listed in PubMed or Embase. Full-text articles were read by two reviewers to identify barriers to surgical task-shifting. Cited barriers were counted and categorized, partly based on the World Health Organization (WHO) health systems building blocks.

**Results:**

Sixty-two articles met the inclusion criteria, and 14 clusters of barriers were identified, which were assigned to four main categories: primary outcomes, NPC workforce, regulation, and environment and resources. Malawi, Tanzania, Uganda, and Mozambique had the largest number of articles reporting barriers, with Uganda reporting the largest variety of barriers from empirical studies only. Obstetric and gynaecologic surgery had more articles and cited barriers than other specialties.

**Conclusion:**

A multitude of factors hampers the provision of surgery by NPCs across SSA. The two main issues are surgical pre-requisites and the need for regulatory and professional frameworks to legitimate and control the surgical practice of NPCs.

## Introduction

Global health has made remarkable gains over the past 25 years, particularly in the area of communicable disease prevention and control [1]. Worldwide, however, five billion people lack access to safe surgical and anaesthesia care, leading to high case fatality rates, even for readily treatable conditions such as acute appendicitis, strangulated hernia, trauma, and obstructed labour [1, 2]. The importance of surgery in the public health agenda is increasingly recognized [1, 3, 4] and received a boost by the 2014 Lancet Commission on Global Surgery which identified gaps in surgical knowledge, policy, and action [1]. Meanwhile, the surgical burden of disease continues to rise, especially in low- and middle-income countries (LMIC), due to a combination of increased metabolic and cardiovascular diseases, cancer, and trauma following road traffic injuries or violence [4, 5]. The burden of unmet surgically treatable diseases points to the need to ensure universal access to safe, essential emergency, and elective surgery [1, 4].

There are major shortages worldwide in surgical workforce [2, 6], especially in sub-Saharan Africa (SSA) which has only 3% of the global health workforce [7] while facing the highest rate of surgical disability-adjusted life years (DALYs) lost, at 38 per 1000 population [3, 4]. Surgical care in SSA is mainly concentrated in urban referral hospitals [1, 5, 7], which have better surgical expertise and infrastructure than rural hospitals [1, 7]. Urban areas are more attractive for specialists, because of better facilities to practise, opportunities for further training, and higher living standards [1, 7]. At district hospitals, the capacity to provide essential surgical care is not always available, partly due to this limited availability of surgical expertise [3]. In several countries, district hospital-level surgery is almost exclusively provided by non-physician clinicians (NPCs), also called clinical officers, associate clinicians, or assistant medical officers. Compared to medical doctors, the duration of surgical training for NPCs is shorter (typically 2 to 3 years), with lower training costs and better retention rates [8, 9]. The term task-shifting applies, which the World Health Organization (WHO) defines as ‘the rational redistribution of tasks from highly qualified workers to health workers with shorter training and fewer qualifications’ [10]. In the case of surgery, it involves the delegation of surgical tasks from surgical specialists or general medical doctors (MDs) to surgically trained NPCs.

A recent review by Federspiel et al. has shown that task-shifting to NPCs is common in SSA as a strategy to increase the surgical, obstetric, and anaesthesia workforce [11]. The authors refer to barriers to surgical task-shifting, but only as a side note. The adoption of national surgical obstetric and anaesthesia plans (NSOAP) in Zambia, Tanzania, Ethiopia, Nigeria, and Rwanda, and the development of such plans in several other countries, alongside several research initiatives to scale up district-level surgery, such as the Clinical Officer Surgical Training in Africa (COST-Africa) and Scaling up Safe Surgery for District and Rural populations in Africa (SURG-Africa) [12], requires identifying and addressing barriers to district-level surgery. While our literature review was conceived to identify barriers to surgical task-shifting, it turned out to be not always possible to disentangle these from barriers to decentralizing surgical care and general barriers to surgery in resource-limited settings. Our article therefore identifies and maps the various barriers to surgery performed by NPCs reported in the literature and analyses how they differ between surgical disciplines as well as between SSA countries.

## Methods

We conducted a scoping review using PubMed and Embase to obtain relevant articles published between January 2000 and May 31, 2018. We included both empirical and non-empirical studies, published in English, French, or Dutch. To formulate a search strategy and guide the selection of inclusion and exclusion criteria, we used a mnemonic that entails the following: Expectation, Client Group, Location, Impact, Professionals, Service (ECLIPSE) and which is particularly suitable to search for health policy and management information [13].

We used the following search terms: (*surgery OR an(a)esthesiology AND sub-Saharan African countries AND (non-physician clinician (AND other synonyms) OR task-shifting*) for Embase and PubMed databases (Additional file 1, S1_Appendix). The query included the various names that are used for NPCs across SSA, such as non-physician provider, clinical officer, assistant medical officer, medical licentiate, and associate clinician. Articles using other terms or referring to expatriate NPCs were included as well. Although we did not focus on a particular surgical discipline, we excluded articles on rarely performed surgeries. Task-shifting could be for both invasive (e.g. Caesarean sections, hernia repairs, hydrocelectomy) and non-invasive (e.g. bone manipulation following fractures) procedures. Although most surgical task-shifting takes place in rural or district level hospitals, we also included articles reporting task-shifting in urban referral hospitals and surgical camps (outreach). Inclusion and exclusion criteria pertained to the client group, type of surgical service, and location.

The initial screening of abstracts was performed by author PH. As the focus of the review is on barriers, we did not explicitly seek articles focusing on enablers to surgical task-shifting; although, in practice, some articles reported both. Authors HB and PH independently reviewed the full text of the included articles. We denoted whether articles described empirical studies or non-empirical studies. Furthermore, we recorded the following parameters of each study: the study setting or country, surgical procedure, surgical discipline, from which surgical specialist to which NPC cadre surgical tasks were shifted, and any barriers to surgery performed by NPCs mentioned. We clustered the latter into distinct categories. In case of discordance, HB and PH reviewed the article once more. If no agreement could be reached this way, author LB was consulted to make the final decision.

## Results

**Fig. 1:**
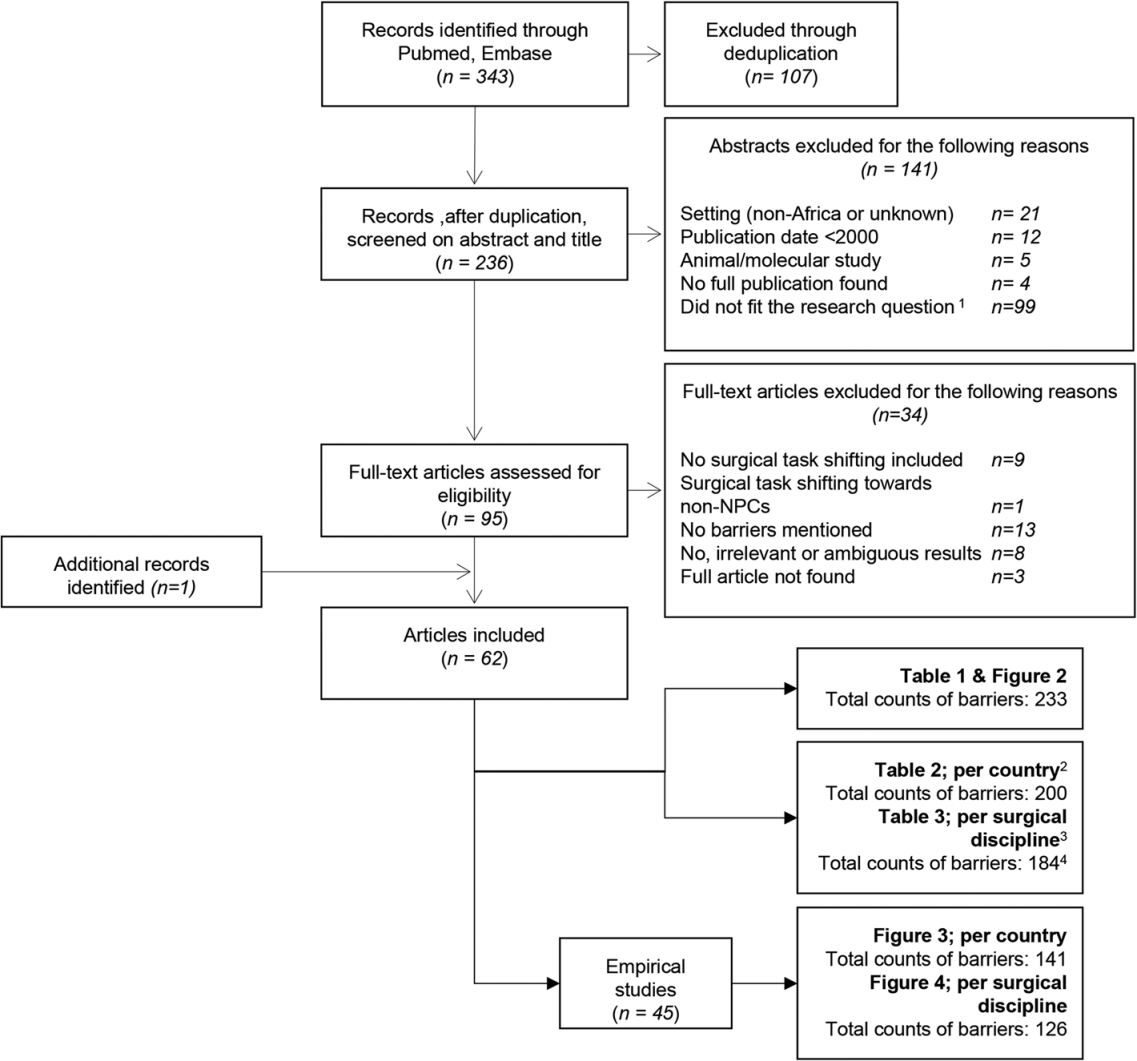
Literature search process and results. Notes: (1) Most common reasons for exclusion of articles are: no mention of NPCs, no mention of task-shifting, no mention of surgery, and merely describing the role of NPCs. (2) Some included articles mention multiple types of barriers per subcategory; hence, de-duplication of barriers to subcategories was performed; thereafter, subcategorized barriers were disaggregated by SSA country. (3) Subcategorized barriers mentioned per article were disaggregated per surgical discipline. (4) Total counts of subcategorized barriers disaggregated by country differ from the total counts of barriers disaggregated by surgical discipline. Articles mentioning multiple SSA countries were counted multiple times, leading to a higher total of counted barriers

Our search resulted in a total of 236 abstracts, after the removal of duplicates. Sixty-two articles met the inclusion criteria (Fig. 1). The majority of these were empirical studies (*N* = 45; 73%). Most of them employed observational/non-interventional methods (*n* = 35), among which 17 comparative studies (nine retrospective, three prospective, four cohort studies, and one case-control study), six descriptive studies (three surveys, two case studies, and content analysis), and 12 interview/focus group studies. There were seven intervention studies, of which six had a before-after design without randomization and one pilot study. The remaining three empirical papers, categorized as ‘other’, included a cost-effectiveness paper, a meta-analysis, and a project evaluation. The non-empirical studies (*N* = 17; 27%) comprised 11 (systematic) literature reviews, one opinion paper, and five ‘other’ descriptive papers.

### Typology of identified barriers

**Fig. 2:**
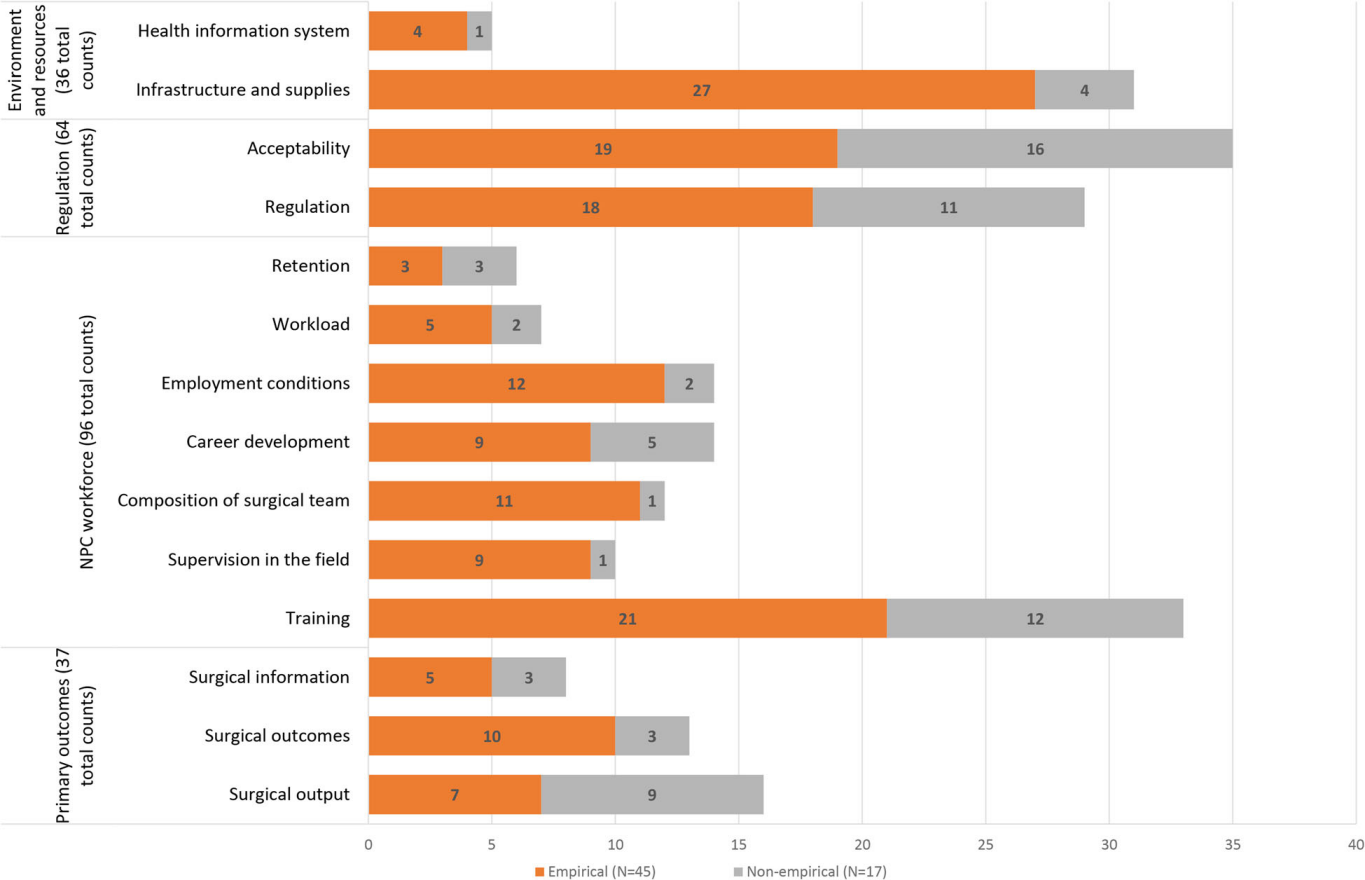
Frequency with which barriers to task-shifting to NPCs are mentioned in the included articles

From the 62 included articles, we identified a total of 233 barriers, of which 160 (69%) were from empirical studies. The barriers were categorized into 14 distinct subcategories, which we further summarized into four main categories of evidence (Additional file 2, S2_Data). We labelled one of these four categories ‘primary outcomes of surgery performed by NPCs’, with a total of 37 barrier counts (Fig. 2) in 28 different articles (Table 1), divided into three subcategories (surgical output, surgical outcomes, surgical information). We labelled the second main category ‘NPC workforce’, divided into seven subcategories (96 barrier counts in 40 articles): training, supervision in the field, composition of surgical team, career development, employment conditions, workload, and retention. The third main category is ‘regulation of surgical task-shifting to NPCs’ (64 barrier counts in 27 different articles), divided into two subcategories: regulation and acceptability. The last category, also subdivided into two subcategories, is ‘environment and resources’ in which NPCs provide surgical services (36 barrier counts in 26 different articles).

**Table 1:** Barriers to surgery performed by NPCs in SSA identified in the literature

Main category of barriers	Sub-category	Type of barrier	Barriers to surgery performed by NPC identified in the literature (***n*** = number of articles mentioning the barrier)	Corresponding references by study types			
				Empirical (***N*** = 45)	References	Non-empirical (***N*** = 17)	References
**I. Primary outcomes** (28 articles)	1. Surgical output	A.1 Range and volume of surgical procedures performed by NPC	1. Inadequate surgical skills *(n = 8)*	(*n* = 3)	[14–16]	(*n* = 5)	[17–21]
			2. Inadequate diagnostic skills *(n = 6)*	(*n* = 3)	[22–24]	(*n* = 3)	[18, 25, 26]
			3. Insufficient opportunities to practise after training *(n = 2)*	(*n* = 1)	[27]	(*n* = 1)	[17]
	2. Surgical outcome	A.2 Surgical outcomes of surgical procedures performed by NPC	4. Low-quality care of NPC, without comparison to MD *(n = 9)*	(*n* = 8)	[15, 28–34]	(*n* = 1)	[18]
			5. Worse surgical outcome compared to MD *(n = 4)*	(*n* = 2)	[35, 36]	(*n* = 2)	[37, 38]
	3. Surgical information	A.3 Availability and quality of information on surgical output/outcomes	6. Ambiguous or incomplete evidence on NPC *(n = 8)*	(*n* = 5)	[27, 31, 36, 39, 40]	(*n* = 3)	[2, 17, 25]
**II. NPC workforce** (40 articles)	4. Training	B.1 Quality and amount of education and training	8. Inadequate pre/in-service training in basic surgical operations *(n = 10)*	(*n* = 8)	[14–16, 30, 35, 41–43]	(*n* = 2)	[18, 44]
			9. Insufficient opportunities for practising during training; inappropriate practice setting *(n = 6)*	(*n* = 3)	[14, 27, 45]	(*n* = 3)	[2, 25, 46]
			10. Inadequate infrastructure or supplies at training facilities *(n = 4)*	(*n* = 4)	[42, 47–49]	-	-
		B.2 Standardization	11. Poor coordination of training *(n = 9)*	(*n* = 4)	[14, 15, 27, 40]	(*n* = 5)	[19–21, 25, 50]
		B.3 Financial support	12. High expenses and inadequate funding for training or education *(n = 4)*	(*n* = 2)	[30, 45]	(*n* = 2)	[2, 8]
	5. Supervision in the field	B.4 Availability of supervision and support for supervision	13. Poor quality of supervision *(n = 2)*	(*n* = 2)	[15, 40]	–	–
			14. Lack of (financial) support and availability of MD for supervision *(n = 8)*	(*n* = 7)	[14, 22, 30, 47, 51–53]	(*n* = 1)	[50]
	6. Composition of surgical team	B.5 Availability of team members	15. Staff shortages *(n = 12)*	(*n* = 11)	[14, 15, 24, 27, 40, 42, 49, 54–57]	(*n* = 1)	[25]
	7. Career development	B.6 Career path	16. Absence of career progression *(n = 10)*	(*n* = 6)	[14, 27, 58–61]	(*n* = 4)	[2, 18, 19, 25]
			17. Behavioural problems due to lack of career progression *(n = 4)*	(*n* = 3)	[14, 30, 59]	(*n* = 1)	[21]
	8. Employment conditions	B.7 Remuneration	18. Insufficient remuneration *(n = 8)*	(*n* = 7)	[14, 27, 30, 54, 58, 60, 62]	(*n* = 1)	[2]
			19. Financial competition between MD and NPC *(n = 2)*	(*n* = 1)	[51]	(*n* = 1)	[8]
		B.8 Prestige, professional status	20. Insufficient professional recognition or status *(n = 4)*	(*n* = 4)	[14, 60–62]	–	–
	9. Workload	B.9 Burden of work	21. High workload *(n = 7)*	(*n* = 5)	[14, 15, 30, 51, 52]	(*n* = 2)	[2, 19]
	10. Retention	B.10 Retention at rural level	22. Difficulties to retain at rural level *(n = 6)*	(*n* = 3)	[22, 30, 47]	(*n* = 3)	[17–19]
**III. Regulation** (27 articles)	11. Regulation	C.1 NPC professional profile	23. Absence of a standardized, legal framework for NPC *(n = 15)*	(*n* = 10)	[14, 30, 33, 34, 40, 41, 51, 52, 58, 63]	(*n* = 5)	[8, 19, 25, 64, 65]
		C.2 Coordination	24. Inadequate coordination and support of NPC practising surgery *(n = 7)*	(*n* = 3)	[27, 30, 40]	(*n* = 4)	[2, 18, 25, 50]
			25. Absence of a regulating body for NPC *(n = 3)*	(*n* = 2)	[58, 61]	(*n* = 1)	[64]
			26. Unsuitable clinical protocols *(n = 4)*	(*n* = 3)	[15, 40, 51]	(*n* = 1)	[64]
	12. Acceptability	C.3 Attitudes of policymakers, health workers, and patients towards NPC	27. General resistance *(n = 16)*	(*n* = 8)	[14, 27, 30, 47, 52, 53, 59, 61]	(*n* = 8)	[2, 8, 19, 25, 46, 64–66]
			28. Fear for loss of power by MD; competition between MD and NPC *(n = 8)*	(*n* = 5)	[22, 27, 34, 52, 59]	(*n* = 3)	[2, 8, 25]
			29. Concerns on quality of care, ethical reservations *(n = 7)*	(*n* = 4)	[14, 30, 52, 59]	(*n* = 3)	[2, 20, 64]
		C.4 Attitude of NPC themselves	30. Negative attitude of NPC *(n = 4)*	(*n* = 2)	[14, 52]	(*n* = 2)	[2, 25]
**IV. Environment and resources** (26 articles)	13. Infrastructure and supplies	D.1 Availability of infrastructure, basic amenities, and equipment	31. Inadequate theatre rooms *(n = 4)*	(*n* = 4)	[27, 47, 52, 57]	–	–
			32. Challenging environmental factors *(n = 4)*	(*n* = 4)	[55, 58, 67, 68]	–	–
			33. Shortages of equipment *(n = 13)*	(*n* = 11)	[15, 22, 30, 40–42, 47–49, 57, 69]	(*n* = 2)	[17, 25]
		D.2 Availability of supplies	34. Supply shortages *(n = 10)*	(*n* = 8)	[30, 40, 47, 48, 54, 55, 63, 69]	(*n* = 2)	[17, 19]
	14. Health information system	D.3 Availability/quality of health information systems	35. Insufficient data recording systems *(n = 5)*	(*n* = 4)	[52, 70–72]	(*n* = 1)	[64]
**Total number of times barriers are mentioned in 62 reviewed articles**				**160**		**73**	

### Primary outcomes of surgery performed by NPCs

Sixteen articles (seven empirical plus nine non-empirical) brought up the limited range and volume of surgical procedures performed by NPCs as a barrier. They report inadequate surgical skills (*n* = 8 articles), with some NPCs practising beyond their abilities [14]; inadequate diagnostic skills (*n* = 6), in respect to the complexity of cases managed by NPCs [25]; and insufficient opportunities to use their surgical skills after their initial training (*n* = 2). Thirteen articles (nine plus four) allude to unsatisfactory outcomes of surgery performed by NPCs, but only four of these actually make a comparison with surgery performed by MDs. Only two of these four articles are based on an empirical study: it involves studies to the outcome of Caesarean sections performed by NPCs, one of them being an audit in Burkina Faso [35] and meta-analyses of several SSA countries [36]. Articles that report surgical outcomes of NPCs (*n* = 9) note multiple instances of poor health outcomes such as high post-operative complication rates following amputation [28] or multiple complications due to inadequate care during or after hydrocelectomy [29]. Eight articles (five plus three) comment on the ambiguous or incomplete evidence about the performance of NPCs as a barrier to surgical task-shifting. In a review of studies on task-shifting of emergency obstetric care (EmOC) to NPCs, several empirical articles show contradictory evidence on the surgical performance of NPCs [25]. A study by Ngcobo et al. in South Africa implies that a simple comparison of male circumcision outcomes between NPCs and MDs is misleading, since the latter handle more complex cases [39].

### NPC workforce

In 25 articles, of which more than half involve empirical studies (*n* = 15 articles), a total of 33 barriers were counted, which are related to NPC training.

Several of them (*n* = 10) report inadequate pre/in-service training. In one of these, interviewed specialists and medical doctors argue that NPC training does not adequately cover surgical theory and clinical practice [14]. With insufficient opportunities and inadequate settings to practise during their training (usually at central hospitals; *n* = 6), NPCs appear not always sufficiently prepared for their future working environment [2]. In-depth interviews with midwives about post-abortion care reveal that insufficient training and lack of experience frustrate NPCs as they may have to refer some of their patients to a physician or to another hospital [15]. Another argument is that little attention is being given to continuous medical education after initial training [30]. Nine articles, of which four involve empirical studies, suggest that NPC training programmes are often not standardized. Related to that, Lobis et al. observe in their mixed-methods study in Malawi and Tanzania that there is confusion among NPCs themselves about which EmOC procedures they are allowed to perform [40]. Other articles, nearly all based on empirical studies, mention difficulties to ensure the supervision of NPCs (*n* = 10). One of the reasons for this is the general shortage of physicians [30], who sometimes prefer more attractive positions in private practice [51]. Two articles argue that surgical supervision is inadequate and sometimes consists of mainly negative feedback [15, 73], eventually leading to job dissatisfaction among NPCs, with some expressing the intention to leave their jobs [73].

Eleven empirical studies (out of a total of 12 articles) point to shortages of specific types of expertise in surgical teams, such as anaesthesia and theatre nursing. An evaluation of Senegal’s task-shifting policy regarding emergency obstetrics argues that the observed rapid increase in numbers and quality of C-sections is highly contingent on the availability of surgical team members [27]. Six articles mention impediments in retaining qualified surgical staff in rural areas, while others mention unattractive employment conditions in general. The latter include high workload (*n* = 7), such as long working hours [51], insufficient remuneration (*n* = 8), poor professional recognition of NPCs (*n* = 4), and limited opportunities for career progression (*n* = 10).

### Regulation of surgical task-shifting to NPCs

Twenty-one articles, mostly empirical (*n* = 13 articles) mention 29 barriers with regard to the regulations and coordination surrounding NPCs. Fifteen articles, of which most are qualitative studies, argue that national legal frameworks are inadequate and that there is a general absence of job descriptions for surgically active NPCs. In Uganda, interviewees indicated that this prevents NPCs from practising surgery for the fear of overstepping legal boundaries and possible litigation [52]. The absence of legal protection and a regulating body for overseeing medical practice (*n* = 3) makes surgical task-shifting, and ultimately clinical governance, challenging [58]. Initially designed as a short-term strategy to relieve the shortage of physicians, a recent literature review suggests that task-shifting has expanded beyond government control in some countries, leaving surgical practice by NPCs unregulated and uncoordinated (*n* = 7) [2]. In addition, clinical protocols are sometimes outdated or not standardized (*n* = 4). Following regulations, even more barriers are mentioned as to the acceptance of NPCs, with 35 barriers counted in 19 articles, of which only half are empirical (*n* = 10). Eight of 16 non-empirical studies report a general reservation among various stakeholders, such as policymakers, medical professionals, and patients, towards NPCs engaging in surgery. Reasons for this include doubts about the competence of NPCs and ethical considerations (*n* = 7), although a fear among medical doctors about loss of status plays a role as well (*n* = 8).

### Environment and resources

Twenty-two articles mention 31 barriers that relate to infrastructure and supplies. Ten of them report shortages of medicines. Failure of health systems to ensure steady supplies is found in a qualitative study in Uganda and an intervention study in Tanzania [30, 54]. A literature review on the success of efforts to scale up eye health suggests that NPCs have limited capacity to negotiate adequate supplies because of their low professional prestige [17]. NPC trainees, in an evaluation study, report that insufficient supplies are an obstacle to surgery at training facilities [47]. In terms of infrastructural barriers, non-functioning or shortages of surgical equipment are most frequently cited (*n* = 13), especially in interviews. This shortage also includes insufficient diagnostic facilities [41]. Several evaluations (*n* = 4) point to inadequate staff accommodation during training, including poor electricity and water supplies. In addition, after their training, NPCs are sometimes deployed at hospitals in remote rural areas with substandard surgical facilities and delays in upgrading them, causing disappointment and frustration [47]. Other empirical studies (*n* = 4) report environmental barriers, such as the Ebola outbreak, that caused a temporary suspension of all NPC training programmes in Sierra Leone [58, 67]. Another environmental factor is the availability and quality of health information systems. Five articles mention insufficient monitoring of surgical service provision as an obstacle (*n* = 5). In-depth interviews and focus groups with clinicians and hospital managers in Uganda elicited their concerns about insufficient documentation of quasi-legal surgical task-shifting to NPCs [52]. The interviewees argued that the routine health management information system would need to capture this. Another barrier is that of insufficient information about what it costs to train NPCs [70]

### Identified barriers disaggregated by SSA countries and surgical disciplines

**Fig. 3:**
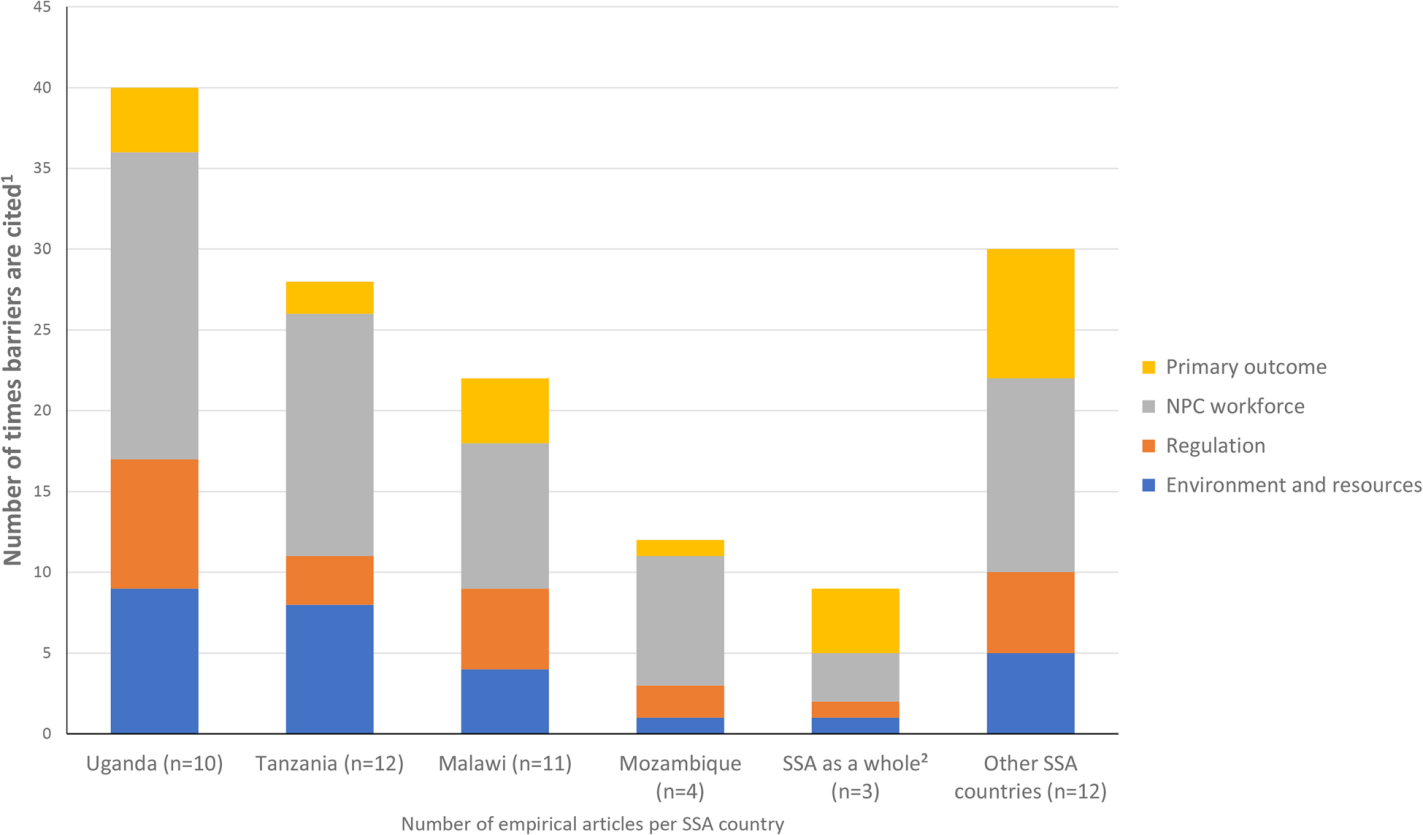
Frequency of barrier subcategories per main category in empirical articles, by SSA country (*n* = number of empirical articles per country). Notes: (1) The main categories represent the sum of all the counts per subcategorized barriers. Hence, some empirical articles might appear more than once per main category if they mention barriers in multiple subcategories. (2) ‘SSA as a whole’ comprises articles about sub-Saharan Africa in general rather than individual countries

**Fig. 4:**
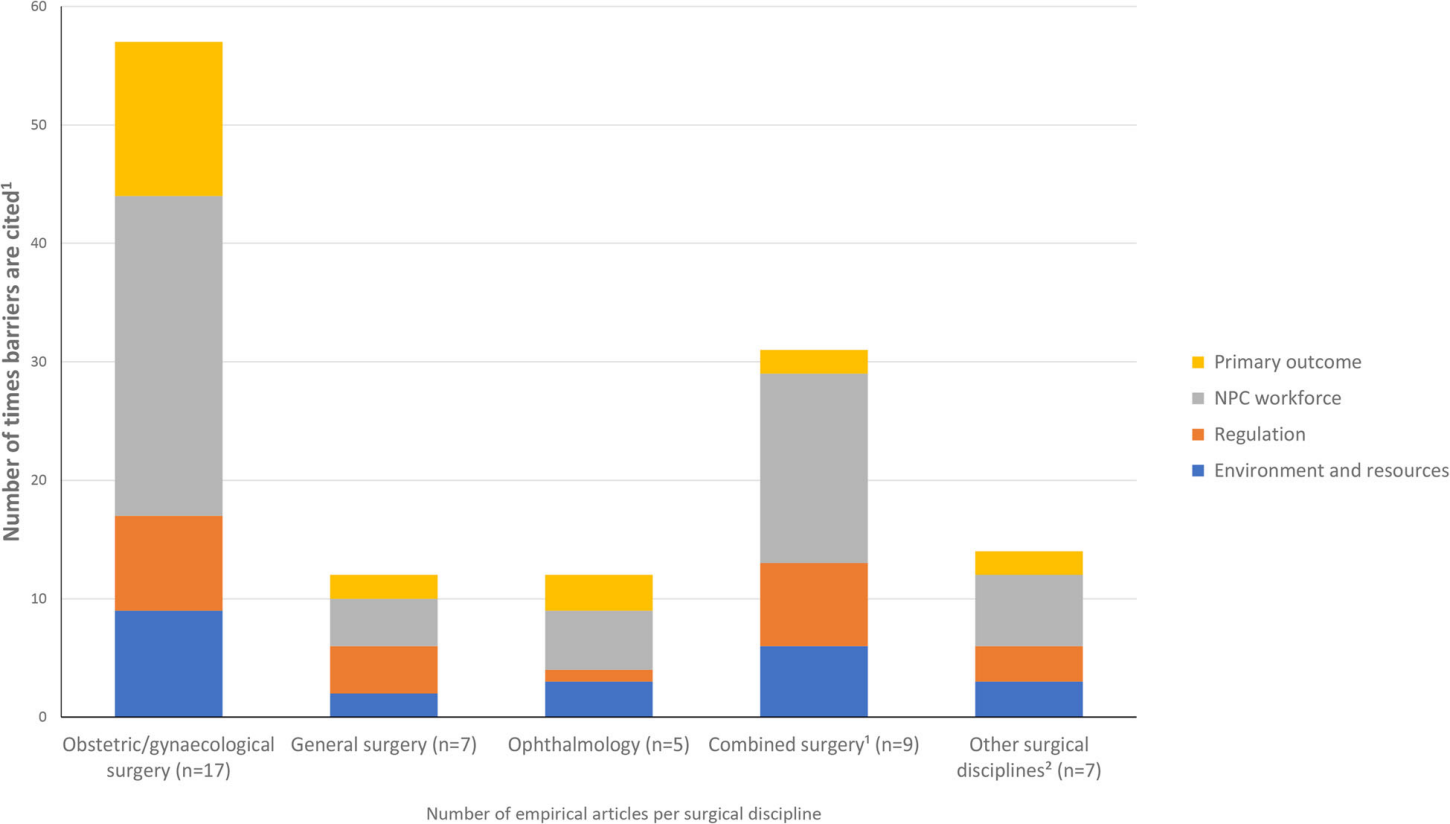
Frequency of barrier subcategories per main category in empirical articles, by surgical discipline (*n* = number of empirical articles per surgical discipline). Notes: (1) ‘Combined surgery’ is when two or more surgical disciplines are described together. (2) ‘Other surgical disciplines’ include orthopaedic surgery (*n* = 2), dermatologic surgery (*n* = 1), emergency surgery (*n* = 1), neurosurgery (*n* = 1), and unspecified surgery (*n* = 2)

We identified 200 barriers spread over different SSA countries (Additional file 3, S3_Table) and 184 barriers spread over multiple surgical disciplines (Additional file 4, S4_Table) In the latter, barriers in obstetric and gynaecological surgery, general surgery, and ophthalmology were the most common in empirical and non-empirical articles combined. Figures 3 and 4 depict the distribution of the four main categories of identified barriers in empirical studies only, by country and by surgical discipline. Malawi (13 articles), Tanzania (13), Uganda (10), and Mozambique (five) have the largest number of articles (Additional file 3, S3_Table). Apart from ‘other SSA countries’, the largest number of barriers per article is in articles involving the ‘SSA region as a whole’ and from Uganda, with 50 and 40 barriers, respectively.

Only studies that took a SSA-wide perspective, which include articles mentioning more than three SSA countries, or use terms such as LMICs in SSA, report on all 14 barrier subcategories. The majority of this category, however, contains non-empirical evidence owing to its wide perspective. In contrast, the articles that focus on individual countries involve relatively more empirical studies. Barriers identified from studies in Tanzania and Uganda are solely from empirical studies, mainly pertaining to the NPC workforce category (Fig. 3)—more specifically their training (with six and five barrier counts)—and the environment and resources category (eight and six barrier counts).

Articles about obstetric and gynaecological surgery (23 articles) cite the largest number of barriers (80 barrier counts in total; see Additional file 4, S4_Table), of which the majority are from empirical studies (Fig. 4). They mainly relate to training, composition of surgical team, and environment and infrastructure and supplies. Ophthalmology, despite being represented by only six articles, has 16 barrier counts, and it is the only discipline for which no regulation barriers are mentioned. Both ophthalmology and general surgery articles (nine articles) show little variation in workforce issues, with no references to barriers in workload or employment conditions.

## Discussion

Our review fills a knowledge gap by identifying and mapping barriers reported in the literature that limit or impact on the surgery performed by NPCs in SSA. It adds to the available evidence on what has been one of the main strategies used in SSA to address surgical workforce shortages in rural areas [74] and sheds light on the evolving roles of NPCs as well as physicians in the region [75].

Two closely intertwined themes are the limitations in national regulation about—often the failure to address—how surgical task-shifting from NPCs to MDs should or should not take place and the often negative perceptions about surgically active NPCs among some policymakers and physicians. Issues in the regulation include a lack of clear mandates and legal protection of NPCs, especially in countries that do not have a formal surgical task-shifting policy and/or legal framework in place [30]. Both of these issues are fuelled to some extent by the question of whether NPCs can deliver optimal or at least acceptable surgical outcomes.

Although some of the articles in our review show positive results of surgeries performed by NPCs [36], there are several that report poor or ambiguous outcomes (Table 1). However, it is important to note that our review was limited to identifying barriers experienced by NPCs doing surgery or other stakeholders. The only studies that compare NPCs with MD in terms of outcomes of surgery performed are an audit trial and a meta-analysis of non-randomized trial results [35, 36]. Wilson et al. observed heterogeneity in the method employed. For instance, while some studies take into account the different circumstances in which NPCs perform operations compared to MDs, other studies do not [36]. Future studies should take such variations into account by performing randomization or by statistically adjusting afterwards. Outside of our review, however, there is some evidence of good or similar outcomes of surgeries performed by NPCs [6]. One example is a recently conducted randomized controlled trial by Gajewski et al., which proved that surgical procedures performed by medical licentiates in Zambia were effective and safe [76]. Furthermore, there is some evidence that surgical task-shifting is actually cost-effective, because of the lower training and deployment costs, among others [2, 62, 64].

The implications of the emerging evidence for surgical task-shifting is a subject of much debate, as also evidenced by the barriers found in this review. On the one hand, drawing on the principles of evidence-based medicine, one could argue that the question of surgical quality needs to be answered more fully, by employing stronger study designs, such as RCTs, before commitments are made by physicians and policymakers to support NPCs doing surgery. This is a position taken by some authors of articles in our review. On the other hand, the reality in many SSA district hospitals is that the alternative to an NPC doing surgery may be that urgent and sometimes life-saving surgery is not done at all or that patients are referred to already distant and over-loaded central hospitals [1, 5, 7]. This can have potentially negative impacts for neglected (often rural) populations through delaying investments into surgical NPC training and deployment, while waiting for definitive evidence on surgical quality.

In any case, if policymakers answer affirmatively to the question whether NPCs should be doing (more) surgery, the question arises of how to achieve this. Our review provides an overview of barriers affecting NPCs doing surgery at the district level that policymakers may need to consider. Apart from the regulatory/acceptance question described above, a large share of the identified barriers relates to the NPC workforce and their training. Our review also demonstrates that training NPCs to do surgery is not sufficient. For surgery to happen, there are other prerequisites such as availability of other surgical team members, infrastructure, and supplies. Furthermore, in order for there to be a sustainable surgical service that serves the needs of the population, the issue of retention at the rural level needs to be addressed through paths such as improved supervision, adequate remuneration, and reasonable workloads. Identifying concrete methods for alleviating or removing barriers was not within the remit of this study. However, in reviewing the articles, we noticed that some do mention such methods. Examples include the use of technological devices which ease surgical procedures for NPCs, such as PrePex in male circumcision [37], or alternative ways of supervision (e.g. telementoring) [16]. In case new RCT studies affirm good outcomes of surgeries performed by NPCs, we would recommend further empirical studies to assess specific technologies or policy interventions allowing NPCs to perform surgery.

This study has several limitations. Firstly, except for the distinction between articles reporting on empirical and non-empirical studies, this review did not evaluate the strength of the evidence or the validity of the identified barriers. The latter implies that we cannot distinguish between barriers that are based on empirical evidence only, interpretations of empirical evidence, and author opinions (which may or may not follow from evidence). Such insights would have required a much more in-depth evaluation of the identified studies. Secondly, we explored the barriers affecting NPCs doing surgery but did not look into enablers such as specific methods, tools, or policy instruments for addressing barriers. Thirdly, we cannot explain why the number of barriers cited in articles varies between countries and between surgical disciplines (as shown in Figs. 3 and 4). There are at least two possible explanations: either there are actually more or greater barriers to surgical task shifting in, for example, Uganda than in a country like Mozambique or in obstetric/gynaecological surgery than in general surgery, or researchers have been more active on surgical task-shifting in Uganda than elsewhere and in obstetric/gynaecological surgery than in other surgical disciplines. The fourth and final limitation is that we did not explicitly look into task-shifting from anaesthesiologists to non-physician anaesthetists, although the provision of safe anaesthesia services is actually a huge challenge in SSA [77, 78], as echoed also by the barrier ‘availability of team members’. We recommend implementation studies to specifically investigate task-shifting of anaesthetic duties and help overcome barriers to anaesthesia provision at the district level.

## Conclusion

While surgical task-shifting to NPCs is widespread in SSA, particularly in rural areas, a multitude of barriers hampers the actual scaling up of surgery, precluding universal access to life-saving and essential elective surgery. In line with a call for the further articulation of global surgery priorities [79], we recommend empirical studies to be undertaken in close collaboration with national health authorities and surgical societies. These should examine the interrelationships between barriers in their local context, as well as how specific interventions might alleviate such barriers, with a view to informing national policies and the development and implementation of surgical, obstetric, and anaesthesia plans. Also, given the large volume of published literature reporting genuine reservations about task-shifting surgical care to NPCs, future research needs to be set in the context of identifying and evaluating feasible, safe, and cost-effective strategies for delivering essential surgery in countries where often surgical specialists and general MDs will not live and work in rural areas.

## Supplementary information

**Additional file 1.** Query as used in PubMed and Embase.

**Additional file 2.** Excel file of counted and categorized barriers.

**Additional file 3.** Frequency with which 14 subcategories of barriers to surgery performed by NPCs are mentioned in empirical and non-empirical articles, by country. Note to table: (1) Some articles describe barriers to surgery by NPCs in more than one country; the total therefore exceeds 62 articles.

**Additional file 4.** Frequency with which 14 subcategories of barriers to surgery performed by NPCs are mentioned in empirical and non-empirical articles, by surgical discipline.

## Data Availability

All data generated or analysed during this study are included in this published article [and its supplementary information files].

## Abbreviations

COST-Africa: Clinical Officer Surgical Training in Africa
C-section: Caesarean section
DALY: Disability-adjusted life years
ECLIPSE: Expectation, Client Group, Location, Impact, Professionals, Service
EmOC: Emergency obstetric care
LMIC: Low- and middle-income countries
MD: Medical doctor
NPC: Non-physician clinicians
NSOAP: National surgical obstetric and anaesthesia plans
RCT: Randomized controlled trial
SSA: Sub-Saharan Africa
SURG-Africa: Scaling up Safe Surgery for District and Rural populations in Africa
WHO: World Health Organization
