# Supplementary material for: Barriers to surgery performed by non-physician clinicians in sub-Saharan Africa—a scoping review
Source: Hum Resour Health. 2020 Jul 17;18:51. doi: 10.1186/s12960-020-00490-y (PMC7368796; doi:10.1186/s12960-020-00490-y)
Supplement: Supplementary file 1 — Additional file 1. Query as used in PubMed and Embase. [file 12960_2020_490_MOESM1_ESM.pdf]

S1 Appendix. Query as used in Pubmed and Embase

Query in Pubmed
(Surg*[tiab] OR anesthe*[tiab] OR anaesthe*[tiab] OR Surgery[MeSH] OR Anaesthesiology[MeSH]) AND ((task*[tiab] AND (shift*[tiab] OR shar*[tiab])) OR non physician clinician*[tiab] OR non physician provider*[tiab] OR Clinical officer*[tiab] OR assistant medical officer*[tiab] OR Medical licentiate*[tiab] OR mid-level practitioner*[tiab] OR associate clinician*[tiab] OR clinical associate*[tiab] OR physician extender*[tiab]) AND (Cameroon* OR Central African Republic* OR Congo* OR Chad* OR Democratic Republic of the Congo* OR Equatorial Guinea* OR Gabon* OR Sao Tome and Principe* OR Burundi* OR Djibouti* OR Eritrea* OR Ethiopia* OR Kenya* OR Rwanda* OR Somalia* OR South Sudan* OR Sudan* OR Tanzania* OR Uganda* OR Angola* OR Botswana* OR Lesotho* OR Malawi* OR Mozambique* OR Namibia* OR South Africa* OR Swaziland* OR Zambia* OR Zimbabwe* OR Benin* OR Burkina Faso* OR Cabo Verde* OR Cote d'Ivoire* OR Gambia* OR Ghana* OR Guinea* OR Guinea-Bissau* OR Liberia* OR Mali* OR Mauritania* OR Niger* OR Nigeria* OR Senegal* OR Sierra Leone* OR Togo* OR sub-Saharan*)

Query in Embase
(Surg*.ti,ab,kw. OR anesthe*.ti,ab,kw. OR anaesthe*.ti,ab,kw. OR surgery/ or general surgery/ OR anesthesiology/) AND ((task*.ti,ab,kw. AND (shift*.ti,ab,kw. OR shar*.ti,ab,kw.)) OR non physician clinician*.ti,ab,kw. OR non physician provider*.ti,ab,kw. OR physician extender*.ti,ab,kw. OR Clinical officer*.ti,ab,kw. OR assistant medical officer*.ti,ab,kw. OR Medical licentiate*.ti,ab,kw. OR mid-level practitioner*.ti,ab,kw. Or associate clinician*.ti,ab,kw. OR clinical associate*.ti,ab,kw.) AND (exp "Africa south of the Sahara"/ OR (Cameroon OR Central African Republic OR Congo OR Chad OR Democratic Republic of the Congo OR Equatorial Guinea OR Gabon OR Sao Tome and Principe OR Burundi OR Djibouti OR Eritrea OR Ethiopia OR Kenya OR Rwanda OR Somalia OR South Sudan OR Sudan OR Tanzania OR Uganda OR Angola OR Botswana OR Lesotho OR Malawi OR Mozambique OR Namibia OR South Africa OR Swaziland OR Zambia OR Zimbabwe OR Benin OR Burkina Faso OR Cabo Verde OR Cote d'Ivoire OR Gambia OR Ghana OR Guinea OR Guinea-Bissau OR Liberia OR Mali OR Mauritania OR Niger OR Nigeria OR Senegal OR Sierra Leone OR Togo OR sub-Saharan).ti,ab,kw.)
