# Supplementary material for: Barriers to surgery performed by non-physician clinicians in sub-Saharan Africa—a scoping review
Source: Hum Resour Health. 2020 Jul 17;18:51. doi: 10.1186/s12960-020-00490-y (PMC7368796; doi:10.1186/s12960-020-00490-y)
Supplement: Supplementary file 3 — Additional file 3. Frequency with which 14 subcategories of barriers to surgery performed by NPCs are mentioned in empirical and non-empirical articles, by country. Note to table: (1) Some articles describe barriers to surgery by NPCs in more than one country; the total therefore exceeds 62 articles. [file 12960_2020_490_MOESM3_ESM.pdf]

## S3 Table. Frequency with which 14 subcategories of barriers to surgery performed by NPCs are mentioned in empirical and non-empirical articles, by country.

| Category | Counted subcategories per article (in empirical ; non-empirical articles) | Uganda (10 articles) | Tanzania (13 articles) | Malawi (9 articles) | Mozambique (14 articles) | SSA as a whole (10 articles) | Other SSA countries (62 articles in total) | Total counts of barrier per category[1] (62 articles in total) |
|---|---|---|---|---|---|---|---|---|
| **I. Primary outcomes** | 1.Surgical output | 1 ; 0 | 1 ; 0 | 1 ; 1 | 1 ; 0 | 2 ; 5 | 1 ; 1 | 14 |
| | 2.Surgical outcomes | 3 ; 0 | - | 2 ; 0 | - | 1 ; 2 | 4 ; 1 | 13 |
| | 3.Surgical information | - | 1 ; 0 | 1 ; 0 | - | 1 ; 2 | 3 ; 1 | 9 |
| **II. NPC workforce** | 4.Training | 5 ; 0 | 6 ; 0 | 2 ; 1 | 1 ; 0 | 0 ; 6 | 3 ; 3 | 27 |
| | 5.Supervision in the field | 4 ; 0 | 2 ; 0 | 2 ; 0 | 2 ; 0 | 1 ; 1 | - | 12 |
| | 6.Composition of surgical team | 2 ; 0 | 5 ; 0 | 2 ; 0 | 1 ; 0 | 1 ; 1 | 3 ; 0 | 15 |
| | 7.Career development | 1 ; 0 | - | 2 ; 1 | 1 ; 0 | 0 ; 3 | 3 ; 1 | 12 |
| | 8.Employment conditions | 2 ; 0 | 1 ; 0 | 1 ; 0 | 2 ; 0 | 0 ; 1 | 3 ; 1 | 11 |
| | 9.Workload | 4 ; 0 | - | - | 1 ; 0 | 0 ; 1 | 0 ; 1 | 7 |
| | 10.Retention | 1 ; 0 | 1 ; 0 | - | - | 1 ; 3 | - | 6 |
| **III. Regulation** | 11.Regulation | 6 ; 0 | 2 ; 0 | 2 ; 1 | 1 ; 0 | 0 ; 6 | 3 ; 1 | 22 |
| | 12.Acceptability | 2 ; 0 | 1 ; 0 | 3 ; 1 | 1 ; 1 | 1 ; 6 | 2 ; 2 | 20 |
| **IV. Environment and resources** | 13.Infrastructure and supplies | 6 ; 0 | 8 ; 0 | 4 ; 0 | - | 1 ; 3 | 5 ; 0 | 27 |
| | 14.Health information system | 3 ; 0 | - | - | 1 ; 0 | 0 ; 1 | - | 5 |
| **Total counts of categorized barriers per country** | | 40 | 28 | 27 | 13 | 50 | 42 | 200 |
