# Supplementary material for: Barriers to surgery performed by non-physician clinicians in sub-Saharan Africa—a scoping review
Source: Hum Resour Health. 2020 Jul 17;18:51. doi: 10.1186/s12960-020-00490-y (PMC7368796; doi:10.1186/s12960-020-00490-y)
Supplement: Supplementary file 4 — Additional file 4. Frequency with which 14 subcategories of barriers to surgery performed by NPCs are mentioned in empirical and non-empirical articles, by surgical discipline. [file 12960_2020_490_MOESM4_ESM.pdf]

S4 Table. Frequency with which 14 subcategories of barriers to surgery performed by NPCs are mentioned in empirical and non-empirical articles, by surgical discipline

Category	Counted subcategories per article (in empirical ; non-empirical articles)	Obstetrics/ gynaecology (23 articles)	Ophthalmology (6 articles)	General Surgery (9 articles)	Combined surgery (14 articles)	Other surgical disciplines (10 articles)	Total counts of barrier per category (62 articles in total)
**I. Primary outcomes**	1.Surgical output	4 ; 2	2 ; 1	0 ; 1	0 ; 2	1 ; 1	14
	2.Surgical outcomes	5 ; 1	1 ; 0	2 ; 1	1 ; 0	1 ; 1	13
	3.Surgical information	4 ; 1	0 ; 1	-	1 ; 1	-	8
**II. NPC workforce**	4.Training	8 ; 5	2 ; 0	1 ; 0	3 ; 4	0 ; 1	24
	5.Supervision in the field	4 ; 1	1 ; 0	1 ; 0	3 ; 0	-	10
	6.Composition of surgical team	7 ; 1	1 ; 0	1 ; 0	1 ; 0	1 ; 0	12
	7.Career development	2 ; 2	-	1 ; 0	2 ; 2	2 ; 1	12
	8.Employment conditions	3 ; 0	-	-	3 ; 2	3 ; 0	11
	9.Workload	2 ; 1	-	-	3 ; 1	-	7
	10.Retention	1 ; 1	1 ; 1	-	1 ; 0	0 ; 1	6
**III. Regulation**	11.Regulation	5 ; 3	-	1 ; 0	5 ; 2	2 ; 3	21
	12.Acceptability	3 ; 3	1 ; 0	3 ; 0	2 ; 4	1 ; 2	19
**IV. Environment and resources**	13.Infrastructure and supplies	7 ; 2	3 ; 1	1 ; 0	5 ; 0	3 ; 0	22
	14.Health information system	2 ; 0	-	1 ; 0	1 ; 0	0 ; 1	5
**Total counts of categorized barriers per country**		80	16	14	49	25	184
